# Dipeptidyl peptidase 1 inhibitors for inflammatory respiratory diseases: mechanisms, clinical trials, and therapeutic prospects

**DOI:** 10.3389/fphar.2025.1656316

**Published:** 2025-08-22

**Authors:** Dandan Zhang, Wenwen Zhang, Ping Hu, Wei Zhang

**Affiliations:** Department of Pharmacy, Shanghai Pulmonary Hospital, Tongji University School of Medicine, Shanghai, China

**Keywords:** dipeptidyl peptidase 1 inhibitors, neutrophil serine proteases, non-cystic fibrosis bronchiectasis, chronic obstructive pulmonary disease, brensocatib, BI 1291583, HSK31858, clinical trials

## Abstract

Dipeptidyl peptidase 1 (DPP1) inhibitors constitute a major advance in respiratory disease therapeutics. Through selective blockade of neutrophil serine protease (NSP) activation, these agents establish novel treatment paradigms for inflammatory respiratory conditions characterized by neutrophil-driven pathology. This comprehensive review examines the development status, clinical efficacy, and safety profile of DPP1 inhibitors in neutrophil-driven diseases, particularly non-cystic fibrosis bronchiectasis (NCFBE) and chronic obstructive pulmonary disease (COPD). Leading compounds including brensocatib, BI 1291583, and HSK31858 have demonstrated substantial clinical efficacy. In the pivotal WILLOW Phase II trial, brensocatib significantly extended time to first exacerbation (hazard ratio 0.58–0.62, p < 0.05) in bronchiectasis patients. The subsequent ASPEN Phase III trial confirmed these findings, with brensocatib reducing annualized exacerbation rates by 21% (10 mg) and 19% (25 mg) compared to placebo (adjusted p = 0.004 and p = 0.005, respectively). Similarly, HSK31858 demonstrated comparable efficacy in Chinese patients, reducing exacerbation risk by 48%–59% in the SAVE-BE trial. While the clinical phenotype observed in Papillon-Lefèvre syndrome (PLS) necessitates careful monitoring of skin and periodontal health during DPP1 inhibition therapy, clinical trials have shown these adverse events occur at low frequencies (1%–4%) and are predominantly mild to moderate in severity. Future research priorities include establishing standardized monitoring protocols for dermatological and periodontal health, developing biomarkers for patient stratification, validating long-term safety profiles, and optimizing combination treatment strategies. With brensocatib potentially becoming the first approved mechanism-specific therapy for bronchiectasis by mid-2025, DPP1 inhibitors represent a paradigm shift in managing neutrophil-mediated respiratory diseases.

## 1 Introduction

Inflammatory respiratory diseases represent formidable public health challenges, substantially diminishing patient quality of life while imposing considerable healthcare and economic burdens (Polverino et al., 2017a; Barbosa and Chalmers, 2023). Despite their heterogeneous etiologies, non-cystic fibrosis bronchiectasis (NCFBE), chronic obstructive pulmonary disease (COPD), and cystic fibrosis (CF) share a fundamental pathological feature: excessive pulmonary neutrophil accumulation with massive release of active neutrophil serine proteases (NSPs) (Korkmaz et al., 2010). These NSPs—neutrophil elastase (NE), proteinase 3 (PR3), and cathepsin G (CatG)—drive persistent lung tissue destruction, abnormal mucus hypersecretion, and sustained inflammatory responses when hyperactivated (Korkmaz et al., 2010; Shoemark et al., 2019).

Current therapeutic approaches emphasize symptom management through bronchodilators, antibiotics, and inhaled corticosteroids, yet these interventions fail to fundamentally alter disease progression (Abo-Leyah and Chalmers, 2017). Traditional anti-inflammatory therapies, particularly corticosteroids, may produce systemic adverse effects while providing suboptimal control of neutrophil-mediated inflammation (Saffar et al., 2011; Wang et al., 2016). The urgent need for innovative targeted therapies addressing core disease mechanisms has become increasingly apparent.

Recently, dipeptidyl peptidase 1 (DPP1) has emerged as a promising therapeutic target, functioning as the critical regulatory enzyme for NSP activation (Chalmers et al., 2025a; Chitsamankhun et al., 2024). NSPs require DPP1 participation during neutrophil maturation to acquire biological activity, and studies confirm that DPP1 inhibition substantially reduces NSP activity, thereby mitigating inflammatory responses and tissue damage (Pham et al., 2004; Guarino et al., 2017; Chalmers et al., 2023). The clinical development of DPP1 inhibitors has progressed remarkably well over the past decade. Several compounds, including brensocatib, BI 1291583, and HSK31858, have completed Phase II and III trials with thousands of patients worldwide, showing reliable effectiveness in reducing bronchiectasis exacerbations across different patient groups (Palmér et al., 2018; Chalmers et al., 2020; Zhong et al., 2025). These consistently positive results from large-scale clinical studies support DPP1 inhibition as a practical therapeutic approach, with brensocatib now nearing regulatory approval. This review examines DPP1 inhibitor mechanisms, clinical research developments, and therapeutic applications, providing systematic insights for clinicians and researchers.

## 2 Biological functions of DPP1 and NSPs

### 2.1 Structural and functional characteristics of DPP1

DPP1 functions as a lysosomal cysteine protease with an approximate molecular weight of 200 kDa, characterized by a distinctive tetrameric architecture. Each monomeric subunit comprises an N-terminal fragment containing the exclusion domain, a heavy chain, and a light chain, which assemble into a stable tetrameric complex through precise intermolecular interactions. The heavy and light chains form the catalytic domain with papain-like folding characteristics, while the N-terminal fragment creates a characteristic β-barrel structure (Turk et al., 2001).

The proenzyme activation process involves complex, highly regulated steps. Initial propeptide removal precedes precise catalytic domain cleavage into heavy and light chains, culminating in fully active tetramer assembly. In the mature DPP1 molecule, the exclusion domain binds the catalytic domain through non-covalent interactions and restricts substrate access to regions upstream of the S2 subsite via steric hindrance, determining DPP1’s unique dipeptidyl peptidase activity (Rebernik et al., 2019a). The exclusion domain creates physical obstruction while its Asp1 residue side chain interacts effectively with substrate N-terminal amino groups, enhancing binding specificity and stability (Rebernik et al., 2019b). This sophisticated architecture enables DPP1 to efficiently activate multiple serine protease precursors, catalyzing the essential conversion from inactive zymogens to biologically active mature enzymes.

The DPP1 active site structure reflects the molecular basis for its broad substrate specificity. The active site features a relatively shallow external pocket (S1 site) and a hydrophobic internal pocket (S2 site), providing structural foundations for processing diverse substrate types. DPP1’s exclusion domain serves as its unique functional component, precisely regulating exopeptidase activity and ensuring specific N-terminal protein substrate processing by restricting substrate entry patterns (Guay et al., 2010).

Regarding cellular distribution, while DPP1 expression occurs across various cell types, expression levels peak in myeloid cells, particularly neutrophils, mast cells, and monocytes (Chalmers et al., 2023). During neutrophil differentiation, DPP1 expression demonstrates distinct spatiotemporal specificity, reaching maximum levels during the promyelocyte stage, coinciding with azurophilic granule formation and establishing the molecular foundation for subsequent NSP activation (Chalmers et al., 2023; Cowland et al., 2016). During this critical developmental period, DPP1 plays an indispensable role in converting NSPs from biologically inert precursors to fully bioactive mature proteases, a transformation essential for normal neutrophil immune function (Gardiner et al., 2016).

### 2.2 Biological functions and pathological significance of NSPs

The NSP family encompasses three key proteases: NE, PR3, and CatG, which serve central roles in neutrophil-mediated innate immunity and inflammatory regulation. Under physiological conditions, NSPs participate in pathogen clearance, extracellular matrix remodeling, and inflammatory response modulation. However, under pathological conditions, excessively activated NSPs become major drivers of tissue destruction, contributing to the development and progression of various inflammatory lung diseases (Korkmaz et al., 2010).

The three primary NSPs exhibit both shared and distinct functional characteristics. NE serves as the principal elastin-degrading enzyme but extends well beyond this role to effectively degrade collagen, fibronectin, and various proteoglycans (Owen and Campbell, 1999). Notably, membrane-bound NE demonstrates enhanced extracellular matrix degradation activity and exhibits resistance to physiological protease inhibitors (Owen et al., 1995). Additionally, NE functions as a potent goblet cell degranulation inducer, promoting excessive mucus secretion and contributing significantly to airway inflammation and chronic obstructive pulmonary disease pathogenesis (Takeyama et al., 1998).

Beyond its proteolytic function, PR3 serves as the major autoantigen in anti-neutrophil cytoplasmic antibody-associated vasculitis, conferring special relevance in autoimmune diseases (Kettritz, 2016). As a highly active serine protease, PR3 can activate multiple important cytokine precursors, including IL-1β and TNF-α, thus playing crucial roles in inflammatory response amplification and maintenance (Metzler et al., 2014).

CatG exhibits distinctive dual biological characteristics, effectively degrading various extracellular matrix proteins while possessing significant antimicrobial activity. Furthermore, CatG participates in complex inflammatory response regulation processes, including cytokine, chemokine, and growth factor processing and activation, as well as apoptosis induction through protease three pathways (Korkmaz et al., 2010).

In pathological states such as bronchiectasis and COPD, NSP activity in sputum and bronchoalveolar lavage fluid increases markedly, with activity levels correlating positively with disease severity, acute exacerbation risk, and lung function decline rates (Shoemark et al., 2019; Chalmers et al., 2017). These findings not only illuminate the central role of NSPs in disease progression but also establish NSP activity as an important biomarker for disease monitoring and treatment evaluation.

### 2.3 Role of DPP1 in NSP activation

DPP1 plays an essential role in activating NSP precursor proteins during neutrophil maturation. This critical biological process occurs primarily during early neutrophil differentiation in bone marrow, concentrated during the promyelocyte to myelocyte transition. DPP1 enables NSPs to acquire complete active conformation and biological function by precisely cleaving specific dipeptide sequences (typically Gly-Glu or Ser-Glu) from NSP precursor N-termini (Méthot et al., 2007).

Recent investigations have identified a “DPP1-like” protease (NSPs-Alternative Activating Protease, NSPs-AAP), adding complexity to NSP activation mechanisms. NSPs-AAP can activate portions of NE and PR3 precursors (approximately 10%) but demonstrates extremely limited CatG precursor activation capacity (<1%) (Adkison et al., 2002; Seren et al., 2018). This alternative activation mechanism explains why DPP1 inhibition affects different NSPs differentially: CatG activity experiences the most significant reduction with DPP1 inhibition, while NE and PR3 retain considerable activity under DPP1 inhibition conditions. This differential inhibition pattern may confer important therapeutic advantages clinically, enabling significant inflammatory damage reduction while preserving partial essential immune defense functions, thereby achieving improved balance between therapeutic efficacy and safety.

## 3 Theoretical basis of DPP1 inhibition strategy

### 3.1 Important insights from Papillon-Lefèvre syndrome

Papillon-Lefèvre syndrome (PLS) provides invaluable theoretical foundations and safety insights for DPP1 inhibition therapeutic strategies. This rare autosomal recessive genetic disorder caused by DPP1 gene (CTSC) mutations occurs with an incidence of approximately 1-4 per million (Toomes et al., 1999). PLS patients experience nearly complete DPP1 function loss accompanied by dramatically reduced NSP activity, clinically manifesting primarily as palmoplantar hyperkeratosis and severe periodontitis. Importantly, these patients do not exhibit severe immunodeficiency (Pham et al., 2004).

This unique clinical phenotype strongly supports DPP1 inhibition strategy feasibility, indicating that even with severely reduced NSP activity, overall immune defense function can remain essentially intact. Neutrophils in PLS patients, while exhibiting some functional abnormalities including decreased chemotaxis, neutrophil extracellular trap (NET) formation defects, and altered pro-inflammatory cytokine release patterns, maintain essentially normal resistance to most common infections (Roberts et al., 2016; Sanchez et al., 2021).

Significantly, detailed studies of PLS patient families provide quantitative reference points for DPP1 inhibitor therapeutic windows. Among PLS patient families, clinically asymptomatic heterozygous carriers demonstrated DPP1 activity reduced to only 13%–47% of controls, suggesting that PLS-like symptoms may only emerge when DPP1 inhibition exceeds 80%–90% (Albandar et al., 2012). This critical finding establishes clear boundaries for safe DPP1 inhibitor application, indicating that moderate DPP1 inhibition may achieve significant anti-inflammatory therapeutic effects while minimizing adverse reactions.

### 3.2 Role of NSPs in inflammatory lung diseases

In inflammatory lung diseases including NCFBE, COPD, and CF, neutrophil-mediated inflammatory responses constitute the core pathological mechanism (Voynow and Shinbashi, 2021; L et al., 2008). These diseases demonstrate significantly increased airway neutrophils with elevated NSP activity (Cantin et al., 2015; Stockley, 1999; Cheetham et al., 2024). Excessive NSPs cause multiple pathological changes: extensive elastic fiber destruction in lung tissue, promoting emphysema formation and progression (Turino, 2007; Stockley); potent airway epithelial cell stimulation, causing excessive mucus production and secretion (Voynow and Rubin, 2009; Fahy and Dickey, 2010); compromised anti-pathogen defense capabilities, creating favorable conditions for bacterial airway colonization and infection (Hartl et al., 2012; King, 2009); activation of multiple pro-inflammatory cytokines, maintaining persistent chronic inflammatory states (Twigg et al., 2015); depletion of endogenous protease inhibitors, further disrupting protease-antiprotease balance (Greene and McElvaney, 2009; Owen, 2008).

Shoemark et al. demonstrated significant correlations between NE activity in bronchiectasis patient sputum and disease severity, bacterial infection status, and acute exacerbation risk (Shoemark et al., 2019). Chalmers et al.’s prospective study further confirmed that elevated sputum NE activity correlates closely with increased future acute exacerbation risk, with high sputum NE activity levels effectively predicting patient disease progression and prognosis (Chalmers et al., 2017). These findings not only reveal NSPs’ pivotal role in disease progression but also establish NSP activity as an important prognostic biomarker (Gramegna et al., 2017).

Traditional NSP-targeted therapeutic strategies primarily involve direct inhibition of activated NSPs, such as the NE inhibitor AZD9668 (Stevens et al., 2011). However, this approach faces numerous limitations: NSPs partially resist inhibitor action after binding to lung matrix (Korkmaz et al., 2010); since multiple different NSPs exist, simultaneous inhibition of multiple NSPs is required for adequate efficacy (Cheetham et al., 2024; Greene and McElvaney, 2009; Owen, 2008); due to extremely high NSP concentrations at inflammatory sites, high local administration concentrations are needed to overcome this challenge (Dubois et al., 2012).

In contrast, DPP1 inhibition strategy theoretically provides more comprehensive anti-inflammatory effects by blocking the upstream NSP activation process, preventing simultaneous activation of multiple NSPs at the source (Palmér et al., 2018). This upstream intervention strategy not only circumvents numerous direct inhibition strategy limitations but may also offer significant advantages in reducing dosing frequency and minimizing adverse reactions.

## 4 Mechanisms of action and pharmacodynamic characteristics of DPP1 inhibitors

### 4.1 Unique mechanism of NSP activation inhibition

**FIGURE 1:**
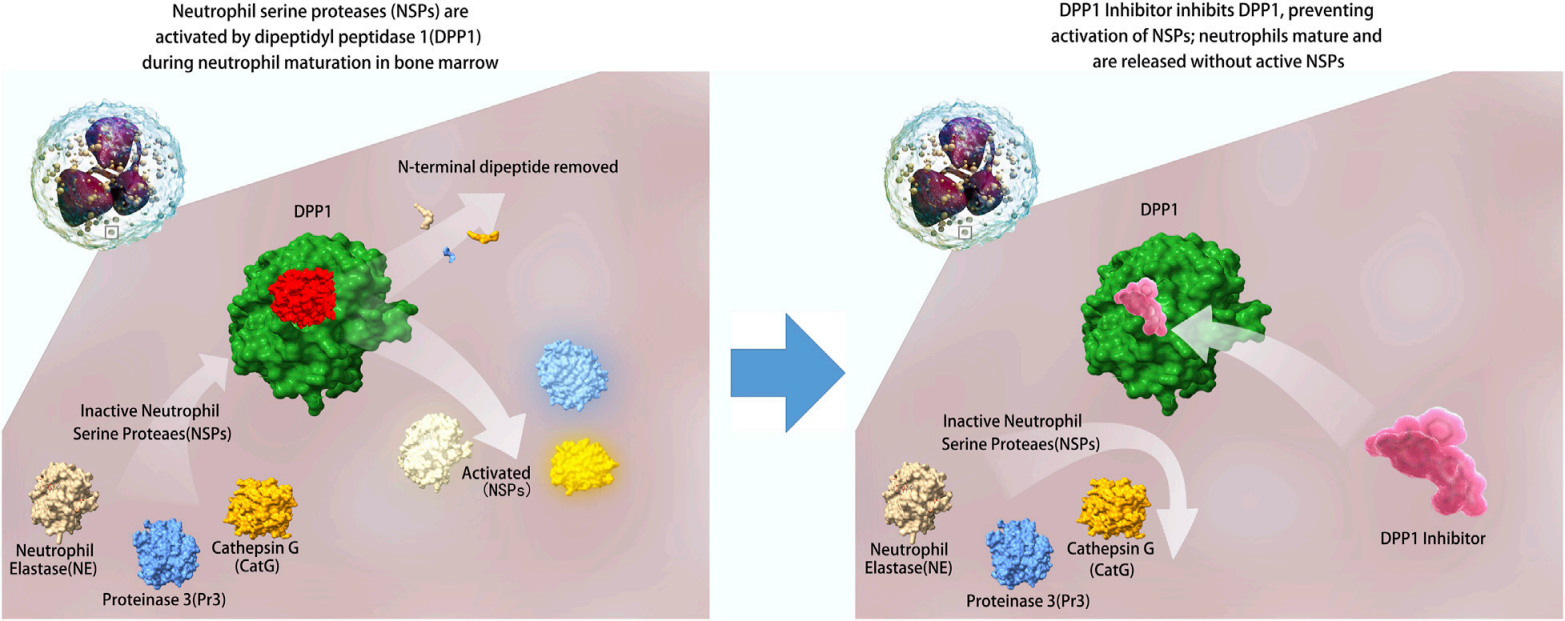
Mechanism of DPP1 inhibition in neutrophil maturation.

The fundamental advantage of DPP1 inhibitors lies in their distinctive mechanism of action: by intervening in the NSP activation process during neutrophil maturation, they prevent accumulation of high concentrations of active NSPs in the lungs. Compared to traditional inhibitors that directly target activated NSPs, DPP1 inhibitors act on the upstream critical link of NSP activation, providing more comprehensive and fundamental inhibitory effects (Korkmaz et al., 2018). The fundamental mechanism by which DPP1 inhibitors exert their therapeutic effects is illustrated in Figure 1.

The therapeutic effects of DPP1 inhibition correlate closely with the physiological neutrophil renewal cycle. In rats, NSP activity inhibition and recovery rates correspond with the 4–6 day neutrophil renewal cycle, with maximum NSP inhibition achieved after 8 days of treatment. Based on human neutrophil renewal rates, DPP1 inhibition is estimated to require approximately 20 days to achieve maximum NSP inhibitory effects in humans (Gardiner et al., 2016). This characteristic indicates that DPP1 inhibitors are not suitable for rapid acute symptom relief but offer advantages for long-term disease management.

Pharmacokinetic studies demonstrate that DPP1 inhibitors such as brensocatib and BI 1291583 can produce sustained and stable NSP inhibitory effects. After oral administration, these drugs can dose-dependently reduce NSP activity in peripheral blood and sputum (Chalmers et al., 2022). Notably, even when drug plasma concentrations decline, NSP activity inhibition can persist for several days, consistent with the physiological process of newly generated neutrophils gradually replacing senescent cells.

### 4.2 Anti-inflammatory effects

NSPs contribute significantly to various inflammatory responses, particularly in lung disease pathological processes, often resulting in destructive cycles of tissue damage and inflammatory responses. Research evidence indicates that DPP1 inhibition can effectively mitigate related inflammatory responses by reducing NSP activity.

In exploratory analysis of the WILLOW study, brensocatib treatment significantly reduced serum NE activity and sputum-related NSP activity, demonstrating broad anti-inflammatory effects (Usansky et al., 2022). Activity of all three major NSPs (NE, PR3, and CatG) in sputum showed significant decreases, and this activity reduction correlated directly with decreased acute exacerbation risk. Simultaneously, inflammatory markers including lactoferrin, IL-8, and IL-1β were correspondingly reduced, further confirming the systemic anti-inflammatory effects of DPP1 inhibition.

Basic research further reveals multiple anti-inflammatory mechanisms of DPP1 inhibition: reduced NET formation, thereby decreasing NET-mediated tissue damage; diminished reactive oxygen species (ROS) production levels, effectively alleviating oxidative stress-induced tissue damage; reduced neutrophil chemotaxis and adhesion capabilities, decreasing inflammatory cell infiltration at lesion sites; decreased excessive mucus secretion, improving airway patency and clearance function.

HSK31858 research results in COPD animal models are equally promising, with this compound demonstrating significant anti-inflammatory activity, effectively reducing inflammatory cell counts and pro-inflammatory cytokine levels in bronchoalveolar lavage fluid (Chen et al., 2021). These extensive and consistent research results strongly suggest that DPP1 inhibition strategies may have important therapeutic value for multiple neutrophil-mediated diseases, establishing new treatment avenues for related conditions.

## 5 Development progress of major DPP1 inhibitors

Multiple DPP1 inhibitors have advanced to clinical research phases, with the most prominent including brensocatib, BI 1291583, and HSK31858. The development progress of these agents provides important clinical validation data for DPP1 inhibition therapeutic strategies.

### 5.1 Brensocatib (AZD7986)

Brensocatib represents an oral, selective DPP1 inhibitor initially developed by AstraZeneca (AZD7986) (Doyle et al., 2016), subsequently acquired by Insmed and advanced through clinical research stages (Chalmers et al., 2022). The drug’s core mechanism involves blocking NSP activation during neutrophil maturation, thereby inhibiting inflammatory mediator release.

Compared to earlier DPP1 inhibitors, brensocatib achieved significant safety improvements, effectively addressing toxicity issues present in previous generation compounds (Doyle et al., 2016). *In vitro* studies demonstrate that brensocatib has nanomolar-level inhibitory activity against human DPP1 while exhibiting excellent selectivity over related proteases such as DPP4 and DPP8/9 (Palmér et al., 2018). This highly selective molecular design establishes brensocatib as a therapeutic agent with excellent clinical application potential.

### 5.2 BI 1291583

BI 1291583 represents a novel DPP1 inhibitor developed by Boehringer Ingelheim, designed to reduce lung NSP activity levels by inhibiting DPP1 activity, thereby restoring the disrupted protease-antiprotease equilibrium in bronchiectasis patients. Preclinical studies indicate that BI 1291583 can bind to human DPP1 in a covalent reversible manner, achieving selective DPP1 enzyme activity inhibition (Kreideweiss et al., 2023).

Regarding pharmacodynamic characteristics, BI 1291583 demonstrates superior inhibitory potency compared to other DPP1 inhibitors. Its half maximal inhibitory concentration (IC50) for NE is 0.7 nM, significantly lower than brensocatib’s 4.0 nM, indicating enhanced inhibitory activity. Additionally, this compound exhibits relatively long *in vivo* half-life characteristics, supporting once-daily dosing regimens and improving patient treatment compliance (Badorrek et al., 2024).

In rodent models, BI 1291583 demonstrated favorable dose-dependent NSP inhibitory effects. Studies show that its NSP activity inhibition degree corresponds with neutrophil physiological renewal rates, achieving maximum inhibitory effects after 8 days of continuous treatment, validating that the compound’s pharmacodynamic mechanisms align with design expectations (Gardiner et al., 2016).

### 5.3 HSK31858

HSK31858 represents a novel oral small-molecule DPP1 inhibitor developed by Haisco Pharmaceutical Group in China (Wang et al., 2025). Unlike the molecular design of brensocatib and BI 1291583, HSK31858 employs a “non-peptide non-covalent” inhibition mechanism, binding to DPP1 through non-covalent interactions with an IC50 of 57.4 nM (Chen et al., 2021).

Structural biology research reveals the unique binding mode of HSK31858 with DPP1. This compound primarily binds to the enzyme’s active site pocket through hydrophobic interactions and hydrogen bond networks but does not form covalent bonds with active site cysteine residues (Chalmers et al., 2023). This non-covalent binding mode may positively influence its inhibition kinetic characteristics and safety profile.

Animal pharmacodynamic studies show that HSK31858-treated mice had NSP activity reduced to 15%–40% of controls and demonstrated significant anti-inflammatory activity in rat COPD models, providing important preliminary validation data for clinical application (Chalmers et al., 2023).

## 6 Clinical research progress of DPP1 inhibitors

### 6.1 Clinical studies of brensocatib

#### 6.1.1 Phase I clinical studies

##### 6.1.1.1 Safety and pharmacokinetics in healthy volunteer

Phase I clinical studies of brensocatib focused on evaluating drug safety, tolerability, pharmacokinetics, and pharmacodynamic characteristics in healthy subjects. Palmér et al. reported Phase I study results in healthy volunteers demonstrating favorable safety and clear dose-dependent pharmacodynamic characteristics under both single and multiple dosing conditions (Palmér et al., 2018). Study data indicated that the drug could significantly inhibit whole blood neutrophil elastase activity, with inhibitory effects showing obvious positive correlation with drug exposure levels. This finding was highly consistent with preclinical model predictions, providing important proof of concept for subsequent clinical studies.

##### 6.1.1.2 Japanese population studies

Another Phase I study in healthy Japanese and Caucasian volunteers further confirmed brensocatib’s pharmacokinetic characteristics. Whether single dosing or multiple dosing after reaching steady state, brensocatib blood exposure showed dose-dependent increases with similar systemic exposure levels between different races. Pharmacokinetic analysis showed brensocatib’s plasma half-life was approximately 24 h, supporting once-daily dosing regimens. Notably, food had relatively limited impact on brensocatib absorption, enhancing clinical application convenience (Usansky et al., 2022).

These early clinical studies successfully established brensocatib’s safe dose range and pharmacodynamic characteristics, providing solid foundations for subsequent Phase II clinical trial design and implementation.

#### 6.1.2 WILLOW study (phase II clinical trial)

##### 6.1.2.1 Study design and population

The WILLOW study represented a pivotal Phase II clinical trial of brensocatib in bronchiectasis patients (Chalmers et al., 2020). This 24-week randomized, double-blind, placebo-controlled, parallel-group trial was conducted at 116 research centers in 14 countries, primarily enrolling non-cystic fibrosis bronchiectasis patients.

The study recruited 416 patients total, with 256 patients meeting inclusion criteria completing randomization, allocated to placebo group (87 patients), 10 mg brensocatib group (82 patients), and 25 mg brensocatib group (87 patients). The primary endpoint was time to first acute exacerbation, with secondary endpoints including acute exacerbation frequency, sputum and serum NSP activity levels, quality of life scores, and safety indicators.

##### 6.1.2.2 Primary efficacy results

Clinical results demonstrated that compared to placebo, once-daily 10 mg or 25 mg brensocatib significantly extended time to first acute exacerbation. The 10 mg group had an adjusted hazard ratio of 0.58 (95% confidence interval: 0.35–0.95, P = 0.03), and the 25 mg group had an adjusted hazard ratio of 0.62 (95% confidence interval: 0.38–0.99, P = 0.046). During the 24-week observation period, risk of any acute exacerbation was reduced by 36% and 25% in the 10 mg and 25 mg groups respectively.

Pharmacodynamic analysis indicated that both brensocatib dose groups showed significant decreases in sputum and serum NSP activity, with inhibitory effects lasting throughout the treatment period. Quality of life scores improved in brensocatib treatment groups but did not reach statistical significance.

##### 6.1.2.3 Pharmacodynamic findings

Further pharmacodynamic analysis evaluated brensocatib’s inhibitory effects on different NSPs (Cipolla et al., 2023). Results showed brensocatib had the most significant inhibitory effect on sputum CatG activity (inhibition rate >90%), with relatively smaller inhibitory effects on PR3 activity (approximately 53%–60%). This differential inhibitory effect is consistent with the aforementioned NSPs-AAP existence mechanism, which can activate portions of NE and PR3 precursors but contributes minimally to CatG precursor activation.

#### 6.1.3 ASPEN study (phase III clinical trial)

##### 6.1.3.1 Study design and scope

Based on positive results from the WILLOW study, Insmed initiated the large-scale Phase III ASPEN clinical trial to further validate brensocatib’s long-term efficacy and safety (National Library of Medicine, 2023a). ASPEN represents a multinational, multicenter, randomized, double-blind, placebo-controlled parallel-group study evaluating clinical benefits of long-term (52-week) brensocatib treatment in non-cystic fibrosis bronchiectasis patients.

##### 6.1.3.2 Primary efficacy results

The ASPEN trial enrolled 1,721 patients across 35 countries to evaluate brensocatib’s effectiveness in bronchiectasis management. The results demonstrated meaningful clinical benefits in reducing exacerbation burden. Patients treated with brensocatib experienced approximately 20% fewer exacerbations annually compared to those receiving placebo. Specifically, the annualized exacerbation rates were 1.02 and 1.04 for the 10-mg and 25-mg doses respectively, *versus* 1.29 for placebo. Both doses achieved statistical significance with p-values below 0.005 (Chalmers et al., 2025b).

The benefits extended beyond exacerbation frequency. Treatment with brensocatib prolonged the time until patients experienced their first exacerbation and increased the proportion who remained exacerbation-free throughout the year-long study. Perhaps most notably, the higher dose showed protective effects on lung function. While all groups experienced some decline in FEV1 over 52 weeks, the 25-mg dose limited this decline to just 24 mL compared to 62 mL with placebo, a difference that reached statistical significance (Chalmers et al., 2025b).

##### 6.1.3.3 Safety profile and tolerability

Throughout the 52-week treatment period, brensocatib demonstrated a reassuring safety profile. The occurrence of adverse events remained comparable across all treatment groups, affecting approximately 77%–80% of participants regardless of whether they received active treatment or placebo. More importantly, very few patients discontinued treatment due to side effects, with rates below 5% in all groups (Chalmers et al., 2025b).

Common adverse events reflected the study’s conduct during the COVID-19 pandemic and typical respiratory symptoms seen in bronchiectasis patients, including COVID-19 infections, nasopharyngitis, cough, and headache. As anticipated from the drug’s mechanism, hyperkeratosis occurred more frequently with brensocatib, particularly at the higher dose. However, these skin changes were generally mild and manageable, resolving without intervention in nearly all cases. Only one patient discontinued treatment due to this side effect (Chalmers et al., 2025b).

The rate of serious adverse events actually trended lower with brensocatib treatment compared to placebo. Notably, despite theoretical concerns about increased infection risk due to neutrophil protease inhibition, no signal for increased severe infections emerged. In fact, pneumonia occurred less frequently in the brensocatib groups than in patients receiving placebo (Chalmers et al., 2025b).

### 6.2 BI 1291583 clinical studies

#### 6.2.1 Phase I clinical study

Phase I clinical studies of BI 1291583 in healthy volunteers demonstrated favorable safety and efficacy characteristics. Study results indicated (Badorrek et al., 2024) that the drug exhibits excellent pharmacokinetic properties and can dose-dependently inhibit NSPs. Whether single or multiple dosing, BI 1291583 could significantly reduce whole blood NE activity, with inhibitory effects closely correlating with drug plasma concentrations.

Pharmacokinetic analysis showed BI 1291583s half-life ranged from 33.6 to 60.2 h, supporting clinical feasibility of once-daily dosing regimens. Based on these positive Phase I study results, the research team established important foundations for Phase II clinical trials of this drug in bronchiectasis patients.

#### 6.2.2 Phase II clinical trials

Based on positive Phase I results, researchers initiated the Phase II clinical trial named Airleaf™ (NCT05238675) in March 2022 (National Library of Medicine, 2025). This represents a multinational, randomized, double-blind, placebo-controlled, parallel-group, dose-exploration study including at least 6 weeks screening period, 24–48 weeks treatment period, and 4 weeks follow-up period. Approximately 322 adult patients with bronchiectasis of various etiologies were randomized to receive once-daily placebo or three different doses of BI 1291583 (1 mg, 2.5 mg, or 5 mg) in a 2:1:1:2 allocation ratio.

Besides the Airleaf™ study, the research team also initiated two additional Phase II clinical trials, Clairafly™ (NCT05865886) and Clairleaf™ (NCT05846230) (National Library of Medicine, 2024) to further explore BI 1291583s application prospects as a potential therapeutic agent for bronchiectasis.

The Clairafly™ trial focuses on evaluating BI 1291583s safety, tolerability, pharmacodynamics, and pharmacokinetics in adult patients with cystic fibrosis-related bronchiectasis (National Library of Medicine, 2024). In this 12-week trial, participants were randomized 2:1 to receive once-daily oral BI 1291583 or placebo.

The Clairleaf™ study represents a long-term safety and efficacy evaluation study specifically designed for bronchiectasis patients who previously participated in Airleaf™ or Clairafly™ trials (National Library of Medicine, 2023b). Participants will receive low, medium, or high doses of BI 1291583 treatment for up to 1 year. These systematic clinical trials will provide key scientific evidence for BI 1291583 as an innovative therapeutic approach for bronchiectasis.

#### 6.2.3 Future development plans

Additionally, the Phase III randomized, double-blind, placebo-controlled clinical trial named AIRTIVITY™ (NCT06872892) is planned to commence in June 2025 (National Library of Medicine, 2022). This trial will evaluate the efficacy, safety, and tolerability of once-daily oral 2.5 mg BI 1291583 for 76 weeks in bronchiectasis patients, providing decisive evidence support for the drug’s final clinical application.

### 6.3 HSK31858 clinical studies

#### 6.3.1 Study design and population

HSK31858 has completed Phase II clinical studies (SAVE-BE) in Chinese bronchiectasis patients (Zhong et al., 2025). This Phase II, randomized, double-blind, placebo-controlled trial was conducted at 25 tertiary medical centers in China, primarily enrolling bronchiectasis patients aged 18 and above who experienced at least two acute exacerbations in the past 12 months. Patients were randomized 1:1:1 to receive 20 mg, 40 mg HSK31858, or placebo treatment, administered orally once daily for 24 weeks.

#### 6.3.2 Primary efficacy results

Study results showed that both doses of HSK31858 could improve clinical outcomes in bronchiectasis patients: Compared to placebo, both the 20 mg group (hazard ratio = 0.52, 95% confidence interval: 0.34–0.80; p = 0.0031) and 40 mg group (hazard ratio = 0.41, 95% confidence interval: 0.26–0.66; p = 0.0002) significantly reduced acute exacerbation frequency. HSK31858 20 mg and 40 mg groups significantly extended mean time to first acute exacerbation.

#### 6.3.3 Safety outcomes

Safety analysis indicated that HSK31858 demonstrated favorable safety and tolerability in Chinese bronchiectasis patients. The proportions of patients experiencing treatment-related adverse events in the three treatment groups (20 mg, 40 mg, and placebo groups) were similar at 86.5%, 88.0%, and 85.3% respectively.

HSK31858s efficacy results were comparable to previous brensocatib and BI 1291583 studies conducted in Europe and America, further validating DPP1 inhibition as an effective strategy for bronchiectasis treatment while providing important clinical evidence for Asian populations.

## 7 Safety evaluation of DPP1 inhibitors

### 7.1 Safety considerations based on PLS patients

Clinical manifestations of PLS patients provide important reference points for safety assessment of DPP1 inhibitors. The main clinical symptoms of PLS patients include palmoplantar hyperkeratosis and severe periodontitis (Sreeramulu et al., 2015). Although these patients have extremely low NSP activity, they do not exhibit obvious immunodeficiency symptoms, providing important theoretical support for the safety of DPP1 inhibition therapeutic strategies (Pham et al., 2004).

Research indicates that neutrophil function in PLS patients shows specific abnormalities, including: reduced chemotaxis, affecting neutrophil directed migration; abnormal pro-inflammatory cytokine release, potentially affecting inflammatory cascade reactions; NET formation defects, potentially affecting clearance of certain pathogens; partially impaired phagocytosis and bactericidal capabilities, but basic functions remain preserved. These abnormalities may partially explain why PLS patients are prone to severe periodontitis (Roberts et al., 2016).

However, notably, PLS patients’ resistance to most common infections usually remains within normal ranges, indicating that basic antibacterial defense mechanisms remain intact (Pham et al., 2004). Although some studies report slightly increased susceptibility to purulent infections in PLS patients, such as skin abscesses and liver abscesses, these patients do not exhibit systemic severe immunodeficiency overall (Sreeramulu et al., 2015).

### 7.2 Safety data from clinical studies

Current studies indicate that both NSP inhibitory effects and skin symptom occurrence require high levels of DPP1 inhibition (Korkmaz et al., 2010; Turk et al., 2001). Clinical manifestations of PLS patients suggest that palmoplantar hyperkeratosis and severe periodontitis occur only when DPP1 function is nearly completely absent, indicating that extremely high levels of inhibition are needed for obvious related clinical symptoms.

In clinical trials of DPP1 inhibitors, all three major DPP-1 inhibitors showed similar safety characteristics, including low incidence rates of skin hyperkeratosis and periodontal events, consistent with the mechanism of action of DPP-1 inhibition. Table 1 summarizes the recent safety characteristics of the three major DPP1 inhibitors (Zhong et al., 2025; Chalmers et al., 2025b; Tadayasu et al., 2025).

**TABLE 1:** Safety data comparison of DPP1 inhibitors.

Safety parameter	Brensocatib (ASPEN study)	BI 1291583 (Japanese phase I)	HSK31858 (SAVE-BE study)
Study Design	Phase 3, 52 weeks	Phase 1, SRD +28-day MD	Phase 2, 24 weeks
Subject Numbers	10 mg: 582; 25 mg: 574	SRD: 18; MD: 9	20 mg: 74; 40 mg: 75
Overall TEAE Rate	10 mg: 77.7%; 25 mg: 76.7%	SRD: 11.1%; MD: 33.3%	20 mg: 86%; 40 mg: 88%
Serious AEs	10 mg: 17.4%; 25 mg: 16.9%	No serious AEs reported	20 mg: 12%; 40 mg: 13%
Skin-Related Adverse Events	Hyperkeratosis: 10 mg: 1.4%; 25 mg: 3.0%	Contact dermatitis: 1 case (5.6%)	Dermatological disorders: 20 mg: 1%; 40 mg: 3%
Periodontal/Gingival Events	10 mg: 1.4%; 25 mg: 2.1%	No oral cavity AEs	20 mg: 4%; 40 mg: 1%
Life-threatening Infections	None reported	None reported	None reported
Drug-related Discontinuation	10 mg: 4.3%; 25 mg: 3.8%	No drug-related discontinuation	20 mg: 0%; 40 mg: 3%
Most Common TEAEs	COVID-19, nasopharyngitis, cough, headache	Contact dermatitis, diarrhea, TMJ syndrome	Upper respiratory tract infections, increased cough, hemoptysis, weight increase

SRD: single-rising-dose.

MD: multiple-dose.

Notably, in various clinical studies, DPP1 inhibitor treatment groups did not show significant increases in serious infection risk compared to placebo groups. This finding is highly consistent with clinical manifestations of PLS patients, supporting the conclusion that DPP1 inhibition has limited impact on host defense functions at therapeutic doses.

### 7.3 Safety monitoring considerations

The skin and periodontal complications associated with DPP1 inhibition present important safety considerations that warrant careful attention. Although we recognize the need for systematic monitoring of these adverse events, the field currently lacks sufficient data to propose specific surveillance strategies.

What we do know is that both dermatological and periodontal manifestations have emerged as consistent findings across studies (Zhong et al., 2025; Chalmers et al., 2025b; Tadayasu et al., 2025). This pattern suggests that clinicians should remain alert to these possibilities when managing patients on DPP1 inhibitors. Early recognition of symptoms could potentially allow for timely intervention, though the optimal management approaches are still being defined.

Patient education plays a crucial role here - ensuring that individuals understand what symptoms to watch for and the importance of prompt reporting. This becomes particularly relevant given that some manifestations may develop gradually and could be overlooked without proper awareness.

Looking ahead, we need prospective studies that systematically track these adverse events to inform evidence-based monitoring recommendations. In the meantime, a prudent approach involves maintaining clinical vigilance and tailoring surveillance to individual patient needs and emerging symptoms. As more data accumulate from ongoing trials, we anticipate being able to offer more concrete guidance on monitoring frequency and specific parameters to assess.

## 8 Clinical application prospects of DPP1 inhibitors

### 8.1 Bronchiectasis

Bronchiectasis represents a chronic respiratory disease characterized by permanent and abnormal bronchial dilation, usually secondary to recurrent airway infections and inflammation (King, 2009). The main clinical manifestations of this disease include persistent cough, sputum production, and recurrent acute exacerbations. These acute exacerbation events not only significantly impact patient quality of life but are also closely associated with progressive lung function decline and increased mortality risk (Chalmers et al., 2014).

Current clinical practice lacks specific therapies approved for treating bronchiectasis, with clinical management primarily relying on empirical treatment strategies including antibiotic use, airway secretion clearance techniques, and bronchodilator application. Among these, long-term use of macrolide antibiotics has been proven to reduce acute exacerbation occurrence risk, but this treatment strategy also brings potential problems of antibiotic resistance and drug adverse reactions (Chalmers et al., 2019). DPP1 inhibitors may offer an effective alternative, particularly for patients seeking to avoid antibiotics due to resistance concerns or those who have not responded well to macrolide prophylaxis. These drugs target neutrophilic inflammation directly, providing a mechanistically different therapeutic approach. Patients with elevated sputum neutrophil counts and NSP activity appear especially well-suited for DPP1 inhibitor therapy instead of standard antibiotics, enabling more tailored treatment decisions based on individual patient characteristics.

DPP1 inhibitors have clear potential advantages in bronchiectasis treatment. They directly target neutrophil-mediated inflammatory responses, which constitute the core pathological mechanism of the disease. They simultaneously inhibit multiple NSPs, providing comprehensive anti-inflammatory effects. By reducing excessive mucus secretion and airway damage, they help improve patient clinical symptoms. They reduce acute exacerbation risk and improve long-term prognosis. The convenience of oral administration helps improve patient treatment compliance.

Brensocatib significantly extended time to first acute exacerbation and reduced acute exacerbation frequency in both the Phase II WILLOW study and Phase III ASPEN study (Chalmers et al., 2020; Chalmers et al., 2025b) HSK31858 achieved similar positive results in the Phase II study (SAVE-BE) in Chinese bronchiectasis patients (Zhong et al., 2025). These study results consistently demonstrate that DPP1 inhibition represents an effective strategy for bronchiectasis treatment.

Insmed has announced plans to rapidly advance brensocatib’s US marketing application, and if approved, it is expected to be marketed in the United States by mid-2025 (Chalmers et al., 2025b). This would potentially provide the first mechanism-specific therapeutic option for bronchiectasis patients.

### 8.2 COPD

COPD represents another therapeutic area with important application potential for DPP1 inhibitors (Singh et al., 2019). NSPs, particularly NE, are closely related to multiple key pathological features of COPD, including inflammatory responses, excessive mucus secretion, and emphysema formation.

Neutrophil-mediated inflammatory responses are considered to play key roles in COPD pathogenesis (Hoenderdos and Condliffe, 2013). COPD patients show elevated NE activity in sputum and bronchoalveolar lavage fluid, with clear correlations to disease severity. Neutrophil counts and NE activity levels are closely related to COPD acute exacerbation risk, lung function decline rates, and disease progression (Barnes, 2016).

Currently, COPD treatment mainly includes bronchodilators, inhaled corticosteroids, and phosphodiesterase-4 inhibitors, but these therapeutic approaches have relatively limited effects on neutrophil-mediated inflammation (Wedzicha et al., 2016). Reducing NSP activity through DPP1 inhibition is expected to significantly improve clinical outcomes in COPD patients.

Although there are currently no large-scale clinical trials of DPP1 inhibitors specifically for COPD, based on positive results in bronchiectasis studies and mechanism of action analysis, DPP1 inhibitors are expected to provide new treatment options for COPD patients, particularly those with neutrophil inflammation as the main characteristic phenotype may achieve more significant therapeutic benefits.

### 8.3 Other potential indications

Besides bronchiectasis and COPD, DPP1 inhibitors have shown important application potential in research for various other indications.

Chronic rhinosinusitis represents one such area. Insmed has initiated a Phase II clinical study of brensocatib for chronic rhinosinusitis with nasal polyps (CRSsNP) (NCT06013241) (National Library of Medicine, 2023c). Chronic rhinosinusitis is also a neutrophil-mediated inflammatory disease (Cho et al., 2016), with pathophysiological characteristics sharing common features with bronchiectasis and COPD.

Cystic fibrosis presents another opportunity. Neutrophil-mediated chronic inflammation represents one of the main pathological features in CF patient airways. In this process, highly active NSPs including NE, CatG, and PR3 are closely associated with disease progression and lung function decline. Theoretically, DPP1 inhibition may improve patient prognosis by reducing NSP-mediated tissue damage and excessive mucus secretion (Mall et al., 2024).

Alpha-1 antitrypsin deficiency represents a genetic disease characterized by reduced alpha-1 antitrypsin levels in the body, where alpha-1 antitrypsin is the main endogenous inhibitor of NE. This imbalance leads to uncontrolled NE activity, subsequently causing early-onset emphysema (Stoller and Aboussouan, 2005). Therefore, DPP1 inhibition may provide a potential new therapeutic strategy for these patients by reducing NE activity.

Acute respiratory distress syndrome (ARDS) involves neutrophil accumulation in alveolar spaces and NSP release, causing alveolar-capillary barrier damage and exacerbated inflammatory responses (Donnelly et al., 1995). Although DPP1 inhibition has slow onset and is not suitable for acute phase ARDS treatment, it may have certain application prospects as a preventive intervention for high-risk populations.

Autoimmune diseases present additional possibilities. In certain autoimmune diseases such as anti-neutrophil cytoplasmic antibody-associated vasculitis, NSPs, especially PR3, participate as autoantigens in disease occurrence and development (Korkmaz et al., 2010). DPP1 inhibition may positively influence disease processes by reducing NSP expression.

## 9 Challenges and future prospects

### 9.1 Current challenges

The development and clinical application of DPP1 inhibitors face multiple challenges that require resolution in future research.

Precise definition of safety windows represents a key challenge. More precise determination of dose ranges that effectively inhibit NSP activity without causing PLS-related symptoms is needed (Miller et al., 2017). Although current research indicates that adverse reactions are controllable at existing doses, more long-term safety data support is still needed to determine optimal treatment regimens.

Appropriate selection of treatment timing presents another consideration. Since DPP1 inhibition effects depend on neutrophil renewal cycles, achieving maximum NSP inhibitory effects in humans requires approximately 20 days (Gardiner et al., 2016). This delay characteristic limits its application value in acute diseases but makes it more suitable for long-term management of chronic diseases. Additionally, NSP activity recovery after drug discontinuation also requires considerable time, presenting special requirements for treatment regimen development.

Potential impacts on immune function require attention. Although PLS patients do not exhibit severe immunodeficiency, the specific impacts of long-term DPP1 inhibitor use on immune function still need further clarification through large-scale long-term clinical studies (Pillay et al., 2010). Particularly in the context of global infectious disease epidemics, closely monitoring these drugs’ effects on anti-infection immunity appears especially important.

Standardization of NSP activity monitoring methods represents a technical need. Since NSP activity is the core pharmacodynamic indicator for evaluating DPP1 inhibition effects, developing simple and accurate NSP activity detection methods is crucial for clinical research and individualized treatment (Chalmers et al., 2022). Currently, methods for measuring NSP activity in sputum and whole blood still require further standardization to improve measurement accuracy and consistency.

### 9.2 Future research directions

Development and validation of biomarkers constitutes a key element for achieving precision treatment. Establishing biomarker systems that can accurately predict DPP1 inhibitor treatment responses is crucial for implementing precision therapy. Existing research has confirmed significant correlations between sputum NSP activity levels and patient clinical outcomes, laying theoretical foundations for their use as predictive markers (Chotirmall and Chalmers, 2024). Simultaneously, in-depth exploration of genetic markers related to DPP-1 inhibitor treatment responses has important clinical value, which will help further refine patient selection criteria and provide scientific basis for individualized treatment.

Exploration of combination treatment strategies deserves investigation. Considering the complex pathophysiological mechanisms of diseases such as bronchiectasis, combination application strategies of DPP1 inhibitors with other therapeutic drugs merit in-depth study (Chalmers et al., 2019). Taking bronchiectasis as an example, DPP1 inhibitors primarily target inflammatory response regulation, while macrolide antibiotics focus on infection control, and their synergistic effects may produce significant therapeutic benefits. WILLOW study subgroup analysis provided preliminary validation of this combination treatment’s clinical value, offering important reference for subsequent research (Chalmers et al., 2025c).

Establishment of long-term safety assessment systems proves essential. Considering potential impacts of DPP-1 inhibitors on skin and oral health, establishing comprehensive long-term safety assessment systems is crucial (Gunsolley et al., 2024). Long-term extension portions of the ASPEN study and other Phase III clinical trials will provide key safety data that will directly influence clinical treatment decisions and provide scientific guidance for optimizing patient management strategies.

Development of novel administration routes shows promise. Development of inhaled DPP1 inhibitors aims to optimize treatment safety and efficacy by directly acting on lung target organs (Boucher, 2019). This administration method is expected to maintain local therapeutic effects while significantly reducing systemic adverse reactions and improving patient treatment compliance and quality of life.

Precision medicine for DPP1 inhibitor therapy hinges on identifying the right patients—particularly those with high sputum neutrophil counts and NSP activity, who tend to respond better to treatment (Sibila et al., 2019). This is where proteomic technologies become particularly useful. Recent work with SELDI-TOF MS and LC-MS/MS has shown we can profile sputum proteins to distinguish between different patient groups and even track how they respond to treatment (Gray et al., 2008; D’Amato et al., 2022). What’s especially interesting is how proteins like calgranulins change during therapy in ways that mirror clinical improvement, which suggests these molecular profiles could help predict who will benefit from DPP1 inhibitors (D’Amato et al., 2022). Rather than relying solely on traditional markers, combining proteomic data with clinical parameters could give us much better tools for selecting patients and monitoring their progress. This integrated approach brings us closer to truly personalized treatment strategies for neutrophil-driven inflammatory diseases.

Development of novel DPP1 inhibitors continues advancing. Next-generation DPP-1 inhibitor development focuses on improving drug selectivity and enhancing safety characteristics. Among these, non-covalent DPP1 inhibitors have attracted considerable attention due to their unique pharmacokinetic characteristics and potential safety advantages (Kreideweiss et al., 2023). Successful development of these novel inhibitors will provide more ideal treatment options for clinical practice, better meeting personalized treatment needs of different patient populations.

## 10 Comparison of DPP1 inhibitors with other treatment strategies

### 10.1 Comparison with direct NSP inhibitors

Direct NSP inhibitors, particularly NE inhibitors such as AZD9668, have undergone Phase II studies in bronchiectasis patients but failed to significantly improve patient clinical symptoms (Stockley et al., 2013). In comparison, DPP1 inhibitors offer several advantages.

Superiority of upstream regulation represents a key advantage. DPP1 inhibitors provide broader anti-inflammatory effects by blocking NSP activation rather than directly inhibiting activated NSPs (Korkmaz et al., 2018). This upstream regulatory strategy can more effectively control inflammatory response cascade processes.

Synergistic effects of multiple targets enhance efficacy. DPP1 inhibition can simultaneously reduce activity of multiple NSPs including NE, PR3, and CatG, while direct inhibitors typically target only single NSPs. WILLOW study exploratory analysis confirmed brensocatib’s inhibitory effects on all three major NSP activities (Cipolla et al., 2023).

Significant improvement in oral administration convenience proves beneficial. Currently developed DPP1 inhibitors are all oral formulations, while many NSP inhibitors require inhalation administration (Polverino et al., 2017b). Oral administration improves patient compliance and convenience of use.

However, DPP1 inhibition also has some potential disadvantages, including slower onset (approximately 20 days to achieve maximum effects) and possible systemic side effects such as hyperkeratosis and periodontitis. In comparison, direct NSP inhibitors have rapid onset but may require more frequent dosing and have limited inhibitory effects on high-concentration NSPs at inflammatory sites.

### 10.2 Differentiated advantages over traditional anti-inflammatory treatment

Compared to traditional anti-inflammatory drugs such as corticosteroids, DPP1 inhibitors provide a more targeted therapeutic approach, which may help reduce systemic side effect occurrence. Although corticosteroids have broad anti-inflammatory effects, long-term use may lead to multiple adverse reactions including osteoporosis, hypertension, and diabetes (Saffar et al., 2011).

Additionally, DPP1 inhibitors may have advantages when dealing with neutrophil-predominant inflammatory responses. Research indicates that corticosteroids have limited effects on promoting neutrophil apoptosis and may even promote their survival in certain circumstances (Wang et al., 2016). Inflammation in diseases such as bronchiectasis and COPD is primarily mediated by neutrophils, and such patients may derive greater benefits from DPP1 inhibition strategies.

### 10.3 Potential for combination treatment

DPP1 inhibitors have potential for combination use with other treatment methods.

Combination with antibiotics shows promise. In bronchiectasis treatment, macrolide antibiotics such as azithromycin have been proven to reduce infectious exacerbation occurrence rates (Al et al., 2013). Considering that DPP1 inhibition can reduce inflammatory responses and bacterial load while improving antibiotic penetration, this combination theoretically could produce synergistic effects.

Combination with bronchodilators presents opportunities. In COPD treatment, DPP1 inhibitors may be combined with bronchodilators, separately targeting inflammation and airway constriction, potentially providing more comprehensive clinical improvement effects (Rogers and Barnes, 2006).

Combination with mucolytic agents offers additional benefits. DPP1 inhibitors reduce mucus production by alleviating inflammatory responses, while mucolytic agents primarily improve clearance efficiency of already produced mucus (Yang et al., 2019). Combined use may produce additive effects in improving airway function, though this hypothesis still requires further clinical validation.

Clinical research preliminarily shows that these combination treatment strategies may provide additional clinical benefits (Yang et al., 2019). In the WILLOW study, some patients simultaneously received macrolide antibiotics and brensocatib treatment, with preliminary results showing potential complementary effects between the two (Chalmers et al., 2025c). However, optimal design of combination treatment regimens and their long-term safety assessment still require more high-quality clinical studies for confirmation.

## 11 Conclusion

The emergence of DPP1 inhibitors brings revolutionary breakthroughs to treatment of respiratory inflammatory diseases. Their uniqueness lies in achieving therapeutic goals by inhibiting NSP activation processes rather than directly antagonizing activated NSPs, establishing entirely new therapeutic avenues for neutrophil-mediated inflammatory respiratory disease management (Barbosa and Chalmers, 2023).

From drug development progress perspectives, brensocatib as the pioneering drug in this field has achieved remarkable results in clinical development. Positive results from the Phase II WILLOW study and Phase III ASPEN study demonstrate that compared to placebo controls, DPP1 inhibition treatment can significantly extend time intervals to first acute exacerbations in bronchiectasis patients and effectively reduce overall acute exacerbation occurrence frequency (Chalmers et al., 2020; Chalmers et al., 2025b). Additionally, other DPP1 inhibitor candidates including BI 1291583 and HSK31858 have shown favorable clinical prospects in their respective development stages, with these research results further validating the enormous potential of DPP1 inhibition strategies as therapeutic approaches for respiratory diseases (Zhong et al., 2025; Badorrek et al., 2024).

From clinical safety analysis perspectives, DPP1 inhibitors demonstrate generally favorable overall safety characteristics. Although theoretically there are potential risks related to skin and periodontal health based on PLS phenotypes, occurrence rates of related adverse events in completed clinical studies have been relatively low, with symptom severity mostly mild to moderate. More importantly, clinical research data have not observed that DPP1 inhibitor treatment significantly increases serious infection risks, supporting the conclusion that DPP1 inhibition has relatively limited impact on host defense functions within therapeutic dose ranges (Zhong et al., 2025; Chalmers et al., 2025b; Tadayasu et al., 2025).

Looking toward future research directions, attention should focus on long-term safety assessment of DPP1 inhibitors, biomarker development, optimal dosage determination, and combination treatment strategies. Through in-depth research in these areas, the therapeutic potential of DPP1 inhibitors can be fully explored, providing more precise and effective treatment regimens for patients.

With steady advancement of regulatory approval processes, DPP1 inhibitors are expected to become important treatment options for diseases such as bronchiectasis in the near future. Simultaneously, their application fields may further expand to COPD, cystic fibrosis, and other neutrophil-mediated inflammatory diseases.

In summary, DPP1 inhibitors establish entirely new therapeutic directions for treatment of respiratory inflammatory diseases, filling gaps in existing treatment approaches and possessing enormous potential for significantly improving patient clinical outcomes and quality of life. As clinical practice experience continues accumulating and basic research continues deepening, this class of innovative drugs is expected to bring clinical benefits to broader patient populations.
